# Supplementary figures and images for: Dissecting cascade computational components in spiking neural networks
Source: PLoS Comput Biol. 2021 Nov 29;17(11):e1009640. doi: 10.1371/journal.pcbi.1009640 (PMC8659421; doi:10.1371/journal.pcbi.1009640)

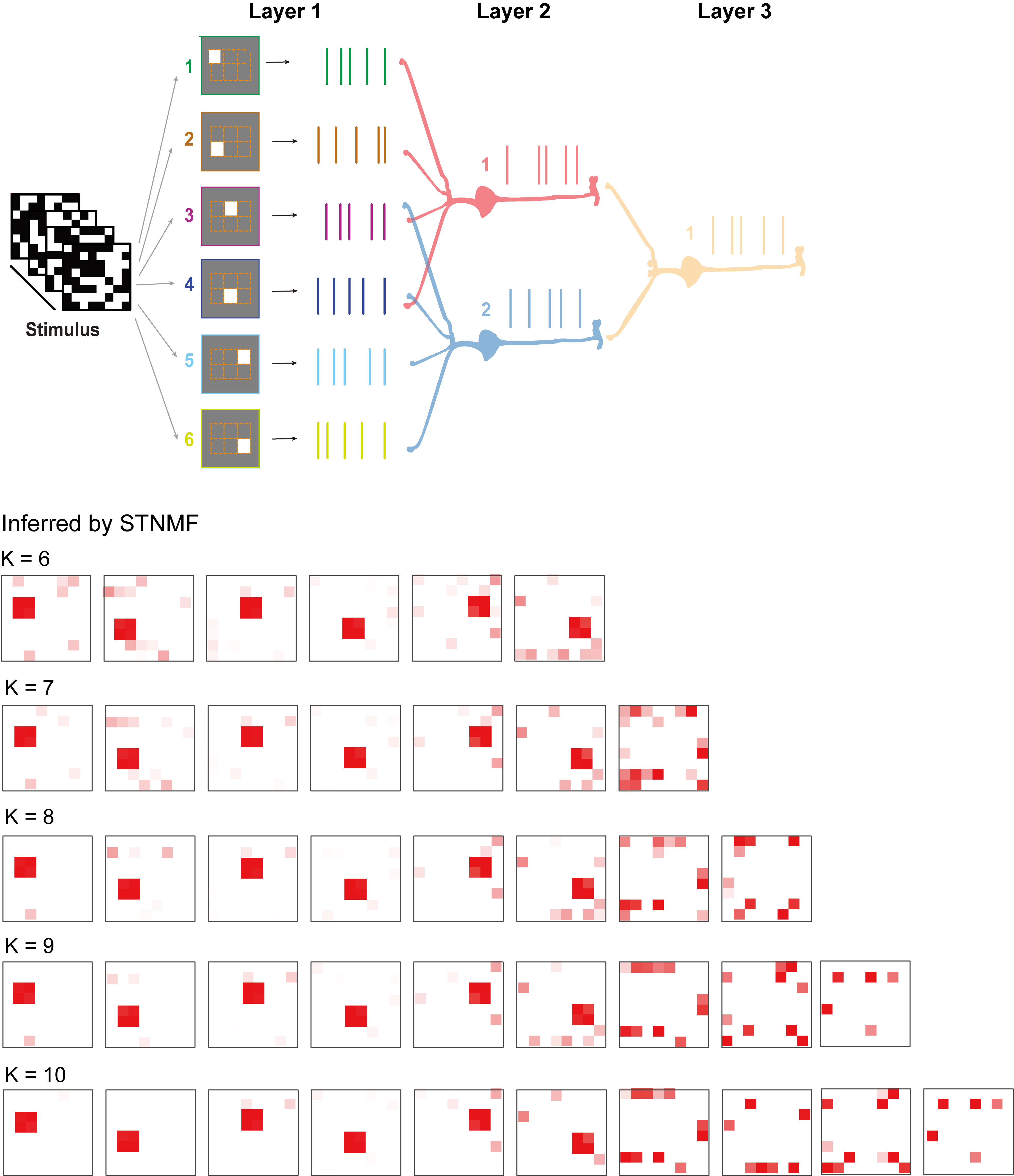

Supplement: S1 Fig — The receptive fields of extra modules are noisy. (TIF) [file pcbi.1009640.s001.tif]

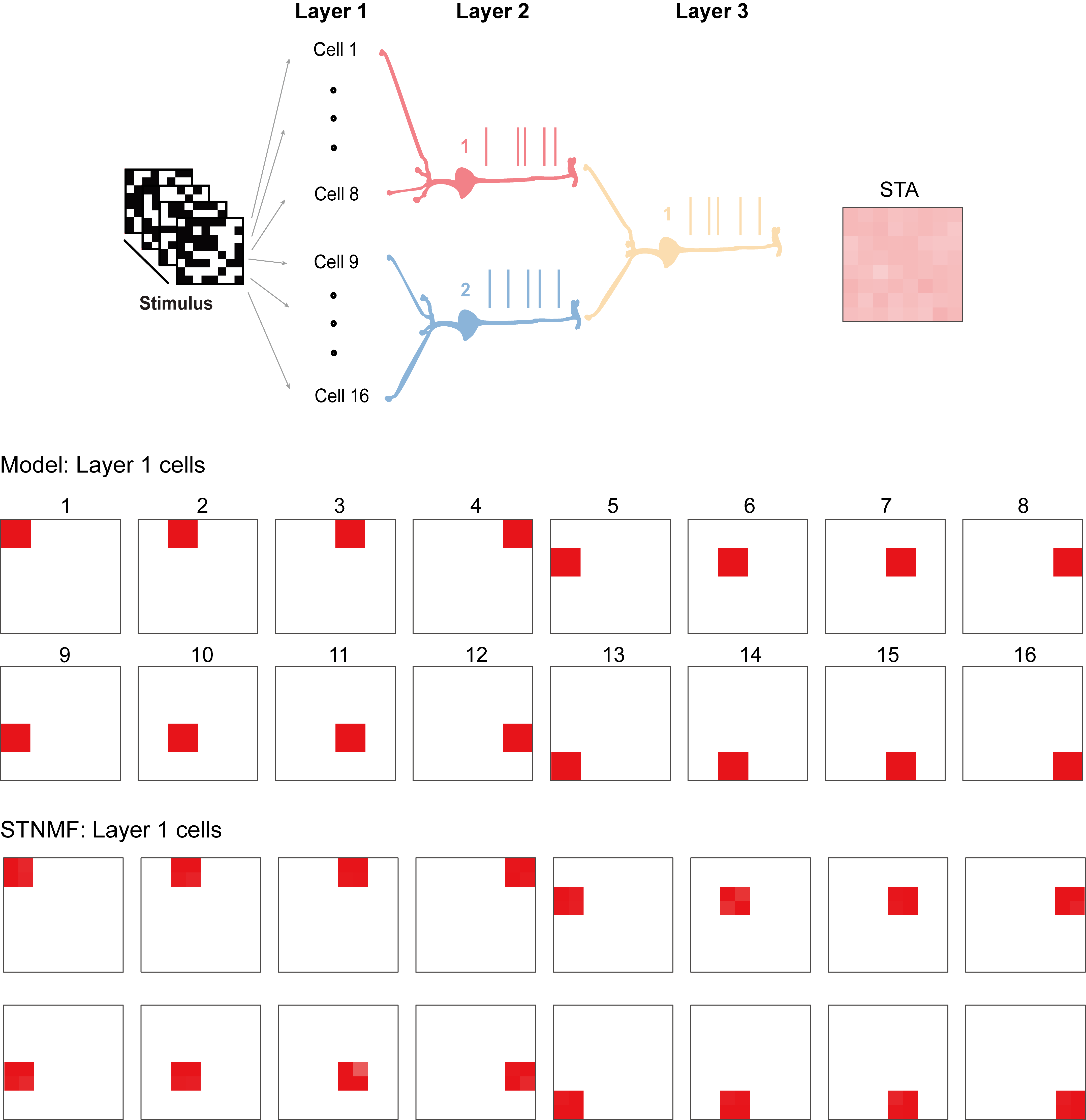

Supplement: S2 Fig — The three-layer network has 16 cells in Layer 1. Among them, neurons 1–8 are connected to the first neuron of Layer 2, and neurons 9–16 are connected to the second neuron of Layer 2. The STA shows the receptive field of the Layer 3 cell. The receptive fields of modeled Layer 1 cells are recovered by the STNMF inference. (TIF) [file pcbi.1009640.s002.tif]

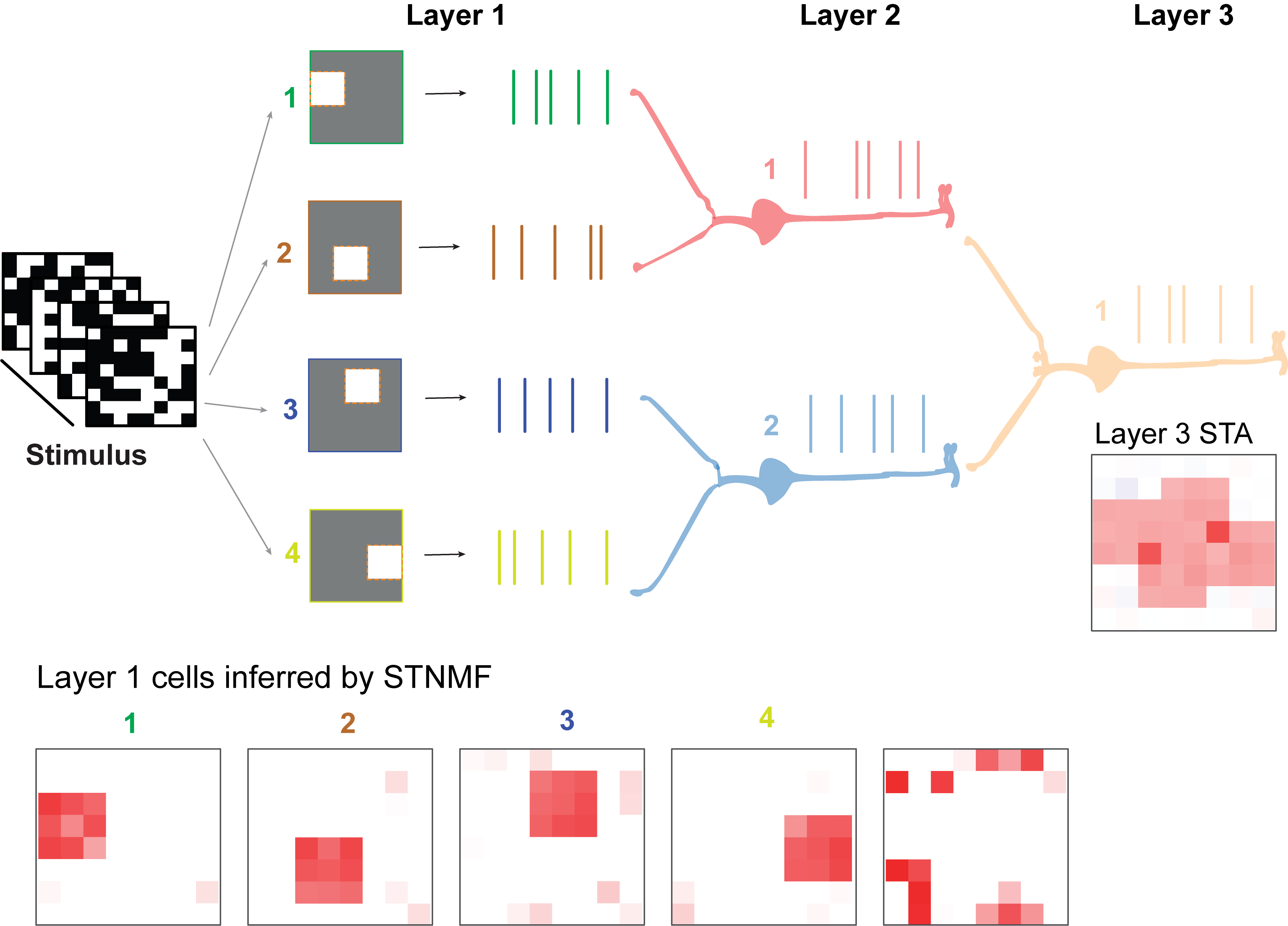

Supplement: S3 Fig — There are four Layer 1 cells with overlapped receptive fields. The STA shows the overall receptive field of the Layer 3 cell, while the STNMF separates them into individual ones of Layer 1 cells. (TIF) [file pcbi.1009640.s003.tif]

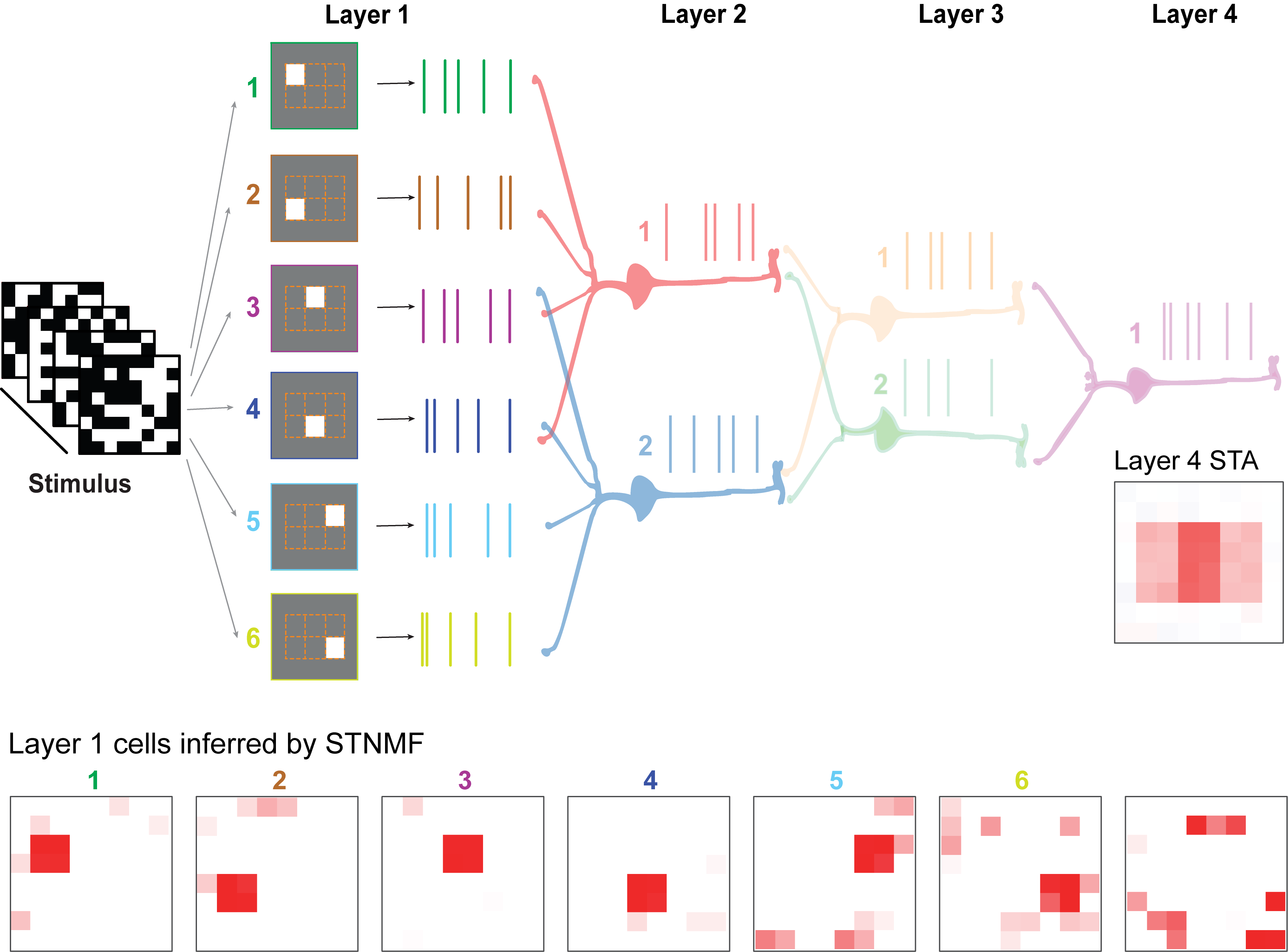

Supplement: S4 Fig — Similar to Fig 3 but with a four-layer structure. The STA shows the receptive field of the Layer 4 cell, while the STNMF obtains the receptive fields of Layer 1 cells. (TIF) [file pcbi.1009640.s004.tif]

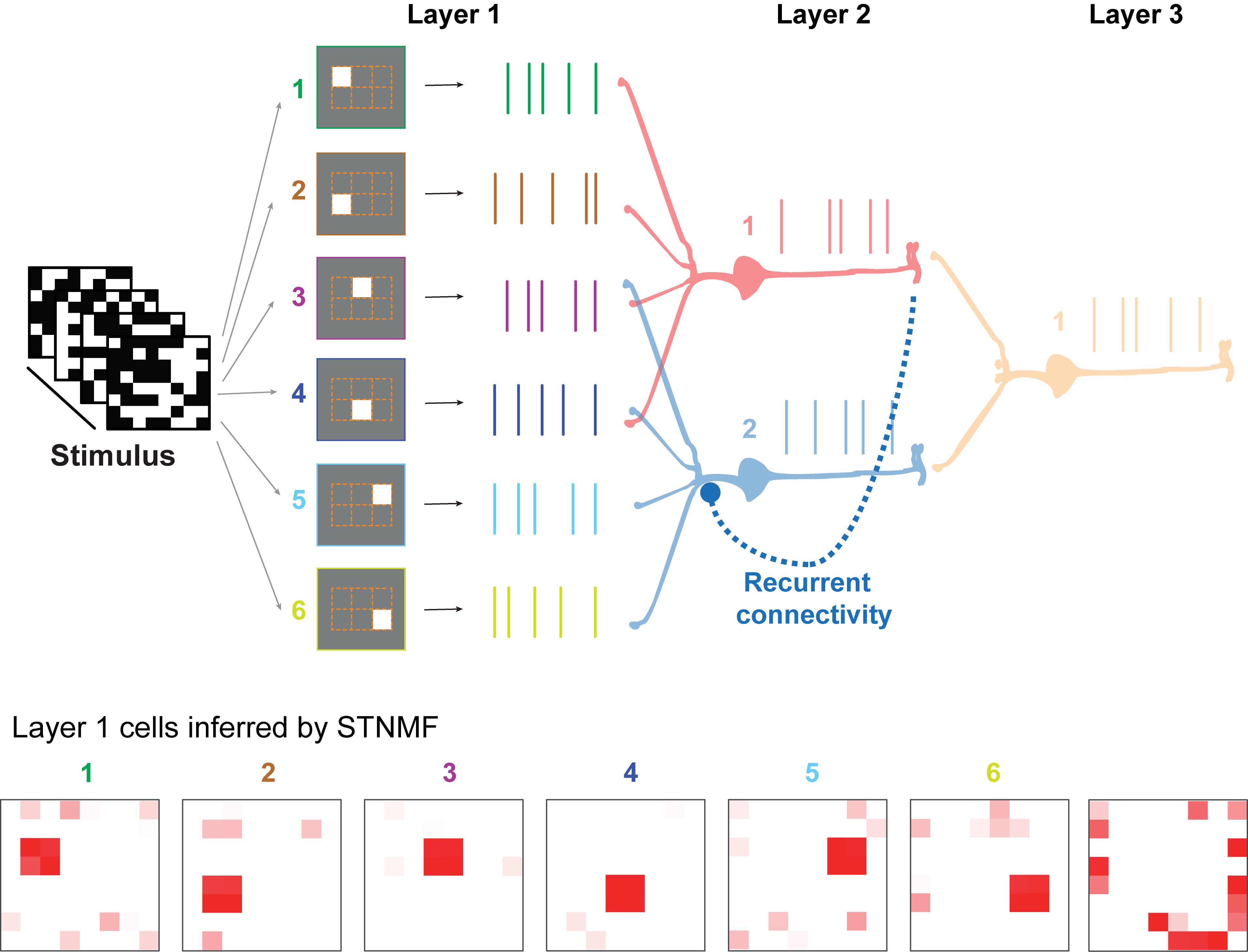

Supplement: S5 Fig — Similar to Fig 3 but with a recurrent connection from the Layer 3 cell to cell 2 in Layer 2. The recurrent connection weight is 0.1, compared to other weights as 1. STNMF can infer the receptive fields of Layer 1 cells. (TIF) [file pcbi.1009640.s005.tif]

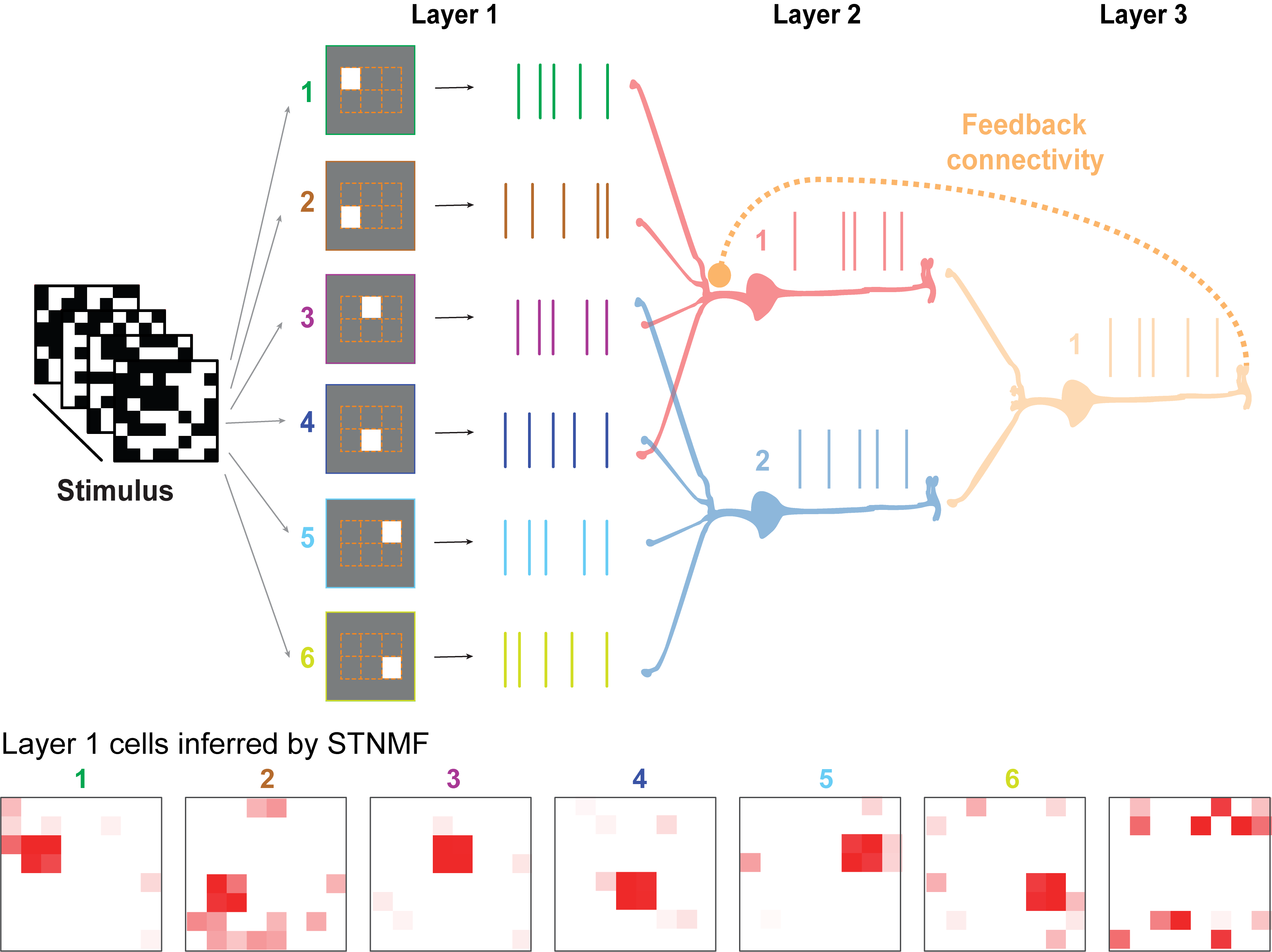

Supplement: S6 Fig — Similar to Fig 3 but with a feedback connection from the Layer 3 cell to cell 1 in Layer 2. The feedback connection weight is 0.1, compared to other weights as 1. STNMF can infer the receptive fields of Layer 1 cells. (TIF) [file pcbi.1009640.s006.tif]
