# Supplementary material for: Dissecting cascade computational components in spiking neural networks
Source: PLoS Comput Biol. 2021 Nov 29;17(11):e1009640. doi: 10.1371/journal.pcbi.1009640 (PMC8659421; doi:10.1371/journal.pcbi.1009640)
Supplement: Attachment — Submitted filename: Response_to_PCB.pdf [file pcbi.1009640.s007.pdf]

PLOS Computational Biology (PCOMPBIOL-D-21-01074)

Dear Editor and Reviewers:

First of all, we would like to express our gratitude to you for carefully reading our manuscript and giving us constructive suggestions. We have carefully read your comments, and revised our manuscript in the light of your remarks and suggestions. You are generous in sharing both your time for carefully reading our manuscript and expertise facilitating our work. Thank you all for your comments during the revision of our manuscript.

The following are point-to-point responses to the reviewers' comments. The reply is arranged in Q's and A's. We first quote your comments, and then give our answers after each of your questions.

****

Replies to the Comments from Editors:

****

In light of the reviews, we would like to invite the resubmission of a significantly-revised version that takes into account the reviewers' comments.

We have carefully revised our manuscript in light of the comments provided by the reviewers. We revised our manuscript significantly to improve the clarity of the main message, added more analysis (newly added Fig.4 and Supplemental Fig. S1-S6), and revised and extended our Discussion (new subsections A and E; revised subsection D).

We provided a version of the revised manuscript with highlights denoting where the text has been revised (in red) for easy reviewing. Please find the marked PDF file uploaded as '**Revised Article with Changes Highlighted.pdf**'. All the responses below indicated by line numbers refer to the marked PDF file rather than the clean copy PDF file.

Thank you very much.

****

Replies to the Comments from Reviewer #1:

****

In the manuscript entitled “dissecting cascade computational components in spiking neural networks”, Jia et. al. exploited a data analysis method named spike-triggered non-negative matrix factorization (STNMF) to infer the dynamical structure of cascade neural networks. The effectiveness of this method has been successfully justified by using the simulation data of multi-layer neural networks in the early visual pathway. As demonstrated by the examples in the manuscript, STNMF is quite effective in extracting the contribution of presynaptic neurons to each spike of a postsynaptic neuron and recovering the connectivity strengths, which will be potentially useful to understand the information coding of feedforward-type neural circuits. The manuscript is already well organized and clearly written, so I only have a few minor comments as listed below.

QI-1. A potential practical problem of STNMF is that one needs to have some prior knowledge about the number of modules/neurons in the first layer, i.e., the parameter K in page 5. But this information is usually hard to obtain. So, is it possible to propose some model selection criteria to help choose an optimal P when applying STNMF?

AI-1: Thanks for the suggestion. In fact, when one uses STNMF to infer the subunits of the network, one does not need to know the parameter K in advance. STNMF has the advantage that the number of modules/neurons will converge as long as K is large enough. To explain this point, we added additional results shown in the newly added Supplemental Fig. S1 with K=6, 7, 8, 9, 10. The analysis results are also as follows.

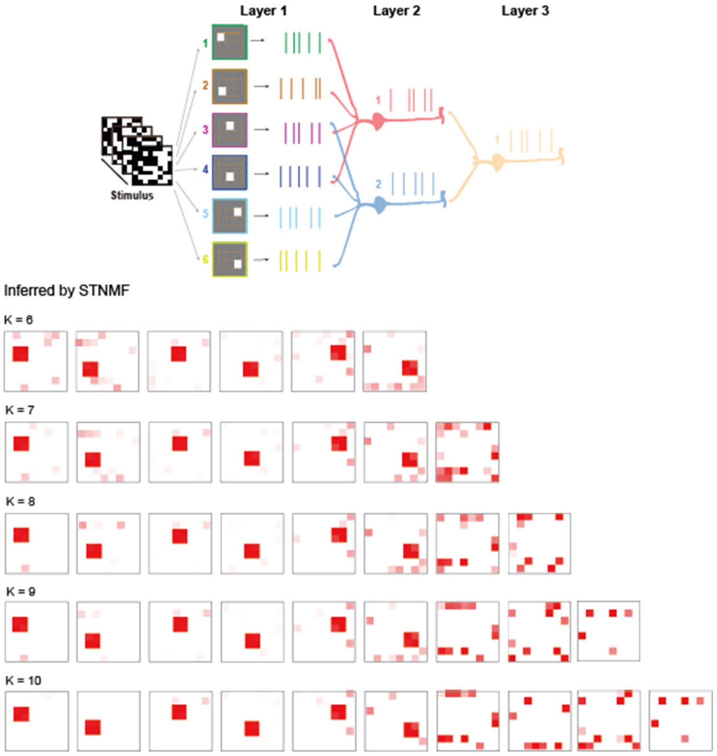

Fig. S1: STNMF analysis with different values of K.

QI-2. It seems that STNMF may be less effective if neurons in the first layer have spatially overlapped receptive fields. Although this case is not fully investigated in the manuscript, some indications can be seen from Fig. 3C right panel. It will be helpful if the authors could discuss a bit on this more realistic scenario.

A2: Thanks for the suggestion. The key component making STNMF work in a neural network is to have the cascade nonlinearity between neurons. In Fig.7 we showed a case with completely overlapped receptive fields mimicking the convergence of a complex V1 cell from LGN cells.

To explain this point better, we considered a three-layer network with 4 overlapped subunits. The STNMF can separate these overlapped subunits. The results are as follows, as well as the newly added supplemental Fig.S3.

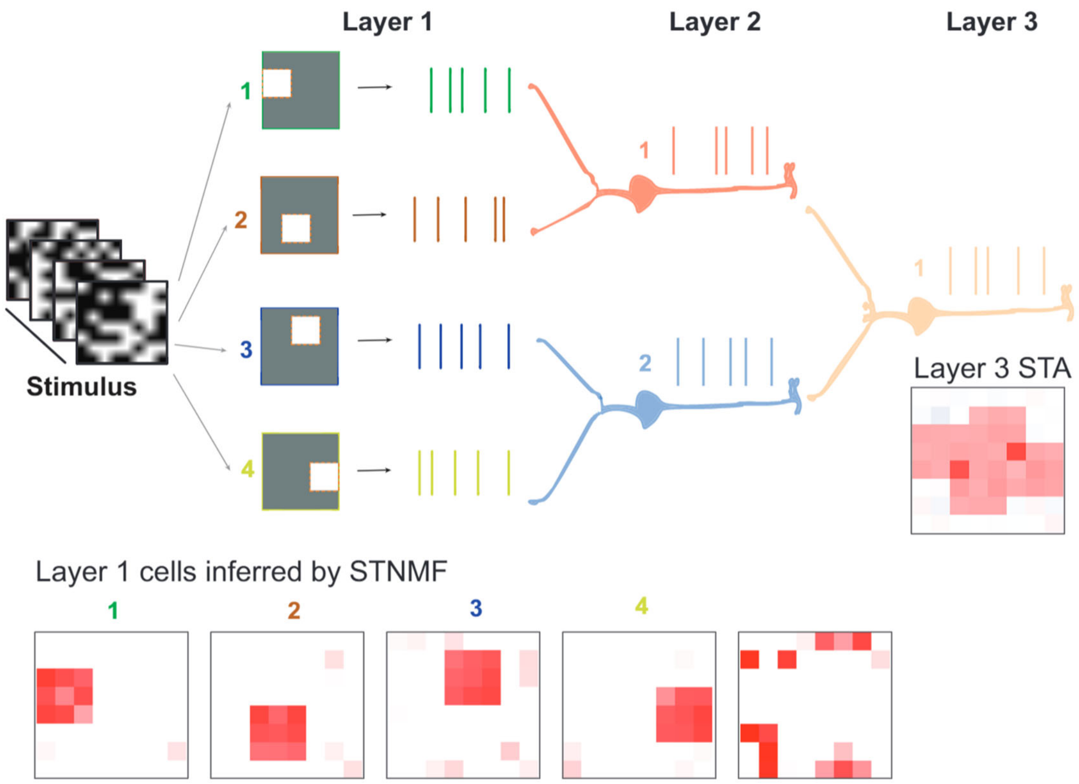

Fig. S3: STNMF analysis with overlapped receptive fields in Layer 1 cells.

QI-3. When inferring the contribution of presynaptic neurons to each spike of a postsynaptic neuron, the corresponding biological interpretation may require more discussion. In general, a resultant spike of a postsynaptic neuron shall attribute to input spikes from multiple presynaptic neurons, not necessarily the case that only one of the presynaptic neurons contributes dominantly. It seems that, when the presynaptic neurons' firing rate is low and the synaptic strength is relatively large, then the inferred spike will be dominantly influenced by a single presynaptic neuron that fires latest. Is the modeling network in this regime?

AI-3: Thanks for the insightful comment. We agree with the Reviewer that biological machinimas are complex and the contribution of presynaptic inputs to a postsynaptic neuron is also complicated. Following the suggestion of the reviewer, we now added

a new subsection in Discussion about this point (read text at line 394: subsec. A. 'The role of presynaptic neurons in postsynaptic neural spikes').

QI-4. Line 92: The math notation r=f(k^Ts(t)) may be misleading. It's better to change it to r=f(k*s(t)), where * represents spatiotemporal convolution.

AI-4: Thanks for the suggestion. We have revised it.

QI-5. Line 104: W_i shall be w_i.

AI-5: We have revised it.

QI-6. Line 106: To be precise, it is better to add a Heaviside function in the expression of the synaptic current, indicating that t starts from t_i^j, and before that time one always has I_syn=0.

AI-6: We have revised it.

QI-7. Line 135: \bar{s}^I shall be defined as either the integral or the summation of the right-hand-side term over \tau.

AI-7: We have revised it.

QI-8. Line 145: Why sparsity is constrained on each column of M rather than each row of M? It is deemed that each row of M is the receptive field of a presynaptic neuron, which needs to be sparsified (because the size of a receptive field is small).

AI-8: Sorry for the confusion. Sparsity was constrained on each column of M due to the mathematical requirement for minimization, see [1]. The sparsity constraint in our case is to control the overall contribution to each spike from a set of modules in each column of M, rather than directly control the size of the receptive field. We have revised this part to make it clear.

[1] Kim, H. & Park, H. Sparse non-negative matrix factorization via alternating non-negativity-constrained least squares for microarray data analysis. SIAM J. Matrix Anal. Appl. 23, 1495–1502 (2007).

QI-9. Line 154, "LNG" shall be "LGN".

AI-9: We have revised it.

QI-10. Line 204, "80100%" shall be a typo.

AI-10: We have revised it.

QI-11. Overall, it is deemed that STNMF is an effective method to infer the dynamical structure of cascade neural networks. In contrast to Granger causality (a popular data analysis method in neuroscience research), STNMF can give more information of the dynamical interaction between neurons. In addition, STNMF may be more applicable to nonlinear neural network dynamics than Granger causality, as Granger causality is proposed based on linear regression models so it can lead to incorrect inference results for neuroscience data, as shown in previous studies:

Stokes P, Purdon P. A study of problems encountered in Granger causality analysis from a neuroscience perspective. PNAS, 114 (34) 7063-7072, 2017.

Li S, Xiao Y, Zhou D, Cai D. Causal inference in nonlinear systems: Granger causality versus time-delayed mutual information. Physical Review E, 97, 052216, 2018.

AI-11: Thanks for the comment. Indeed, Granger causality has been widely used for neural networks and network science. STNMF utilizes the nonlinearity of neural dynamics for inference and may be more applicable to neural networks. Further work is needed to explore the difference and compare these methods, and we are happy to work on this and report it later. We now cited these two useful references and added some discussion about this part (red text at line 419).

****

Replies to the Comments from Reviewer #2

****

Reviewer #2: The manuscript "Dissecting cascade computational components in spiking neural networks" by Jia et al., has employed a recent method, termed spike-triggered non-negative matrix factorization (STNMF) to extract the functional connections in a set of simulated two- and three-layer feedforward network models. The results proved that STNMF method could be able to successfully derive the connectivity structure and provide predictions of synaptic weights. The results are solid and interesting and I like the STNMF method, while there are several concerns/flaws make it hard to be convinced for the PLoS Comp Biol. journal.

QII-1. strictly speaking, the STNMF method was developed in authors' previous publication (ref.17), and already was applied in a two-layer feedforward network model (ref.27), hence the present manuscript (with add results of 3-layer network) does not contain enough novelties. Also, there are a lot of repeated results/ statements were already done in previous works.

AII-1: Here we showed a new application domain, multiple-layer and fully spiking neural networks, with STNMF, while the previous works were not about spiking neural networks. To further demonstrate the utility of STNMF in fully spiking neural networks, we have now added a series of networks with different scenarios, including more cells in layer 1 (Supplemental Fig.S2), 4-layer network (Supplemental Fig.S4), networks with recurrence (Supplemental Fig.S5) or feedback (Supplemental Fig.S6), and a complex scenario using natural image stimulus (new Fig.4) that is a hard question for any other existing methods.

The methodology of STNMF was introduced previously, as noted by the reviewer, where the applicable context was the specific neural circuitry of the retina. Specifically, the STNMF was considered in the restricted two-layer network of bipolar cells and ganglion cells, where there are no spikes in bipolar cells as the first layer. Thus in previous work, there was no direct evidence that the STNMF can work for a full spiking neural network with spiking neurons at every layer.

In the current manuscript, we provided evidence that STNMF is applicable to fully spiking neural networks with multiple layers. As noted by the reviewer, we

demonstrated various scenarios with 3-layer networks. Since there is a significant difference between 2-layer and 3-layer networks, as evidenced by the classical studies of artificial neural networks, adding a hidden layer in the 2-layer network can dramatically expand the computation power of a neural network. Similarly, inferring cascade computations in a 3(or more)-layer network is not trivial for neural circuitry. To our knowledge, this has not been studied in the literature. The only exception case was a study of the retinal neural circuitry [1], where the input layer (layer 1) of photoreceptors can be inferred using the neural activity of the output layer (layer 3) of ganglion cells. However, this method is restricted and limited to the scenario of macaque retina only. The STNMF was introduced and shown validated for the 2-layer bipolar cell-ganglion cell network of the retinas of salamander and mouse. The significance of the present manuscript is that we showed the STNMF is applicable to multiple (3 and more)-layer fully spiking neural networks.

We also revised the text to avoid repeated statements, and made our description more concise.

[1] Functional connectivity in the retina at the resolution of photoreceptors. Field et al. Nature 467 (7316), 673-677 (2010).

QII-2. second, although authors claimed several times in the manuscript that the STNMF method could be a general approach for neural systems with kinds of functional connectivity structures. However, it may be overstated since this paper as well as their previous works only show that it works well for feedforward-type of networks, but no evidence for networks with feedback or other re-current connectivities.

AII-2: Thanks for the comment and sorry for the overstatement. We have revised our description and avoided overstatement.

STNMF could be a useful approach for neural networks. However, similar to other methods, such as Granger causality mentioned in **Q1-11/A1-11**, there are certain limitations as well. STNMF is working well for feedforward networks, as well as for networks with weak feedback or recurrence. It is generally believed that there are many types of connections in neural systems, and feedback and recurrence are existing in neural circuitry as part of the connectome or structural connection. In some cases, even there is strong structural recurrence in the network, the dynamics and/or the function of the network could change the synaptic strengths to make the network more feedforward, e.g., a functional forward information flow in a recurrent network, typical neural trajectories in song birds or hippocampus studies. Since STNMF is a method for inference of the functional connection using neural activities/spikes, it could be possible that STNMF is still working for the networks with feedback/recurrence. Together with the points discussed in **Q1-3/A1-3**, the spare coding principle and local community properties of neural networks could make STNMF work within a certain regime of conditions. We have revised the text to avoid overstatement. We also added a new subsection of Discussion about these discussions (red text at line 497 'E. Limitations').

To consider the feedback and recurrence in the network, we conducted two new sets

of analyses using a neural network model with weak feedback or recurrence.

1) Feedback. We added a feedback connection from the layer 3 neuron to one layer 2 neuron. The feedback connection weight was set to 0.1, compared to other feedforward weights as of 1. STNMF can obtain the similar results as shown below, as well as the newly added Supplemental Fig. 6.

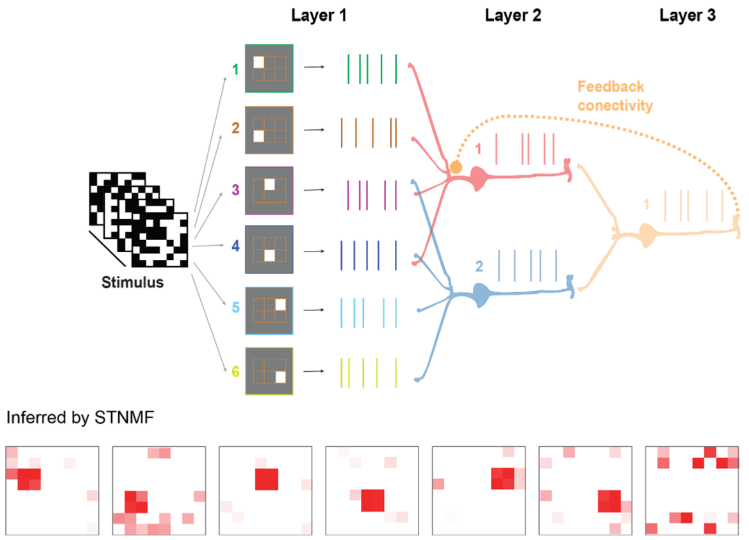

Fig. S6 Feedback network.

2.) Recurrence. We added a recurrent connection on a layer 2 cell, where the output of this cell fed into its own input. The connection weight was set to 0.1, compared to other connection weights as of 1. STNMF can obtain the similar results as shown below, as well as the newly added Supplemental Fig. 5.

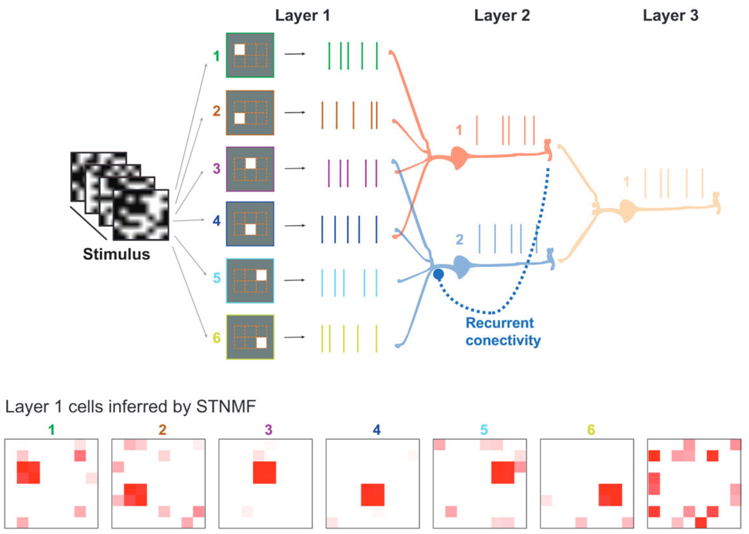

Fig. S5 Recurrent network

We found STNMF works well with weak feedback/recurrence. Stronger feedback/recurrence in the current setting defer STNMF. Future work is needed to explore this, as discussed above and in the new subsection (red text at line 497 'E. Limitations'). We have added these results into the supplemental materials, and revised the text to make our statements more clear.

QII-3. indeed if only apply the method for the simulated network models, authors could have more open directions to extend the STNMF method, for example, this manuscript could consider over the white noise-type stimuli, since previous work by Jack Gallant group showed that colored noise-type stimuli could be better one to detect the neurons' receptive field.

AII-3: Thanks for the comment. Indeed, compared to white noise stimuli, colored noise and natural scenes are more efficient to trigger neural responses. The receptive field was traditionally well defined with simple white noise stimuli. The advantage of white noise is that it has no spatial correlation, so all the inferred receptive field information comes from the characteristics of neurons. It is well known that the receptive field is not static but dynamic when using complex stimuli. The spatial correlation of complex stimuli, colored noise, or natural scenes, introduces additional complexity. Actually, how to define the receptive field using natural scenes is still an open question. But we agree with the reviewer that the demonstration of the STNMF with complex stimuli is very interesting.

For this, we designed a simulation using natural images as stimuli. We used a large set of natural images from ImageNet. The analysis of STNMF shows that the receptive field of layer 1 cells can still be found as shown below and in newly added Fig.4 (more details can be found at line 318).

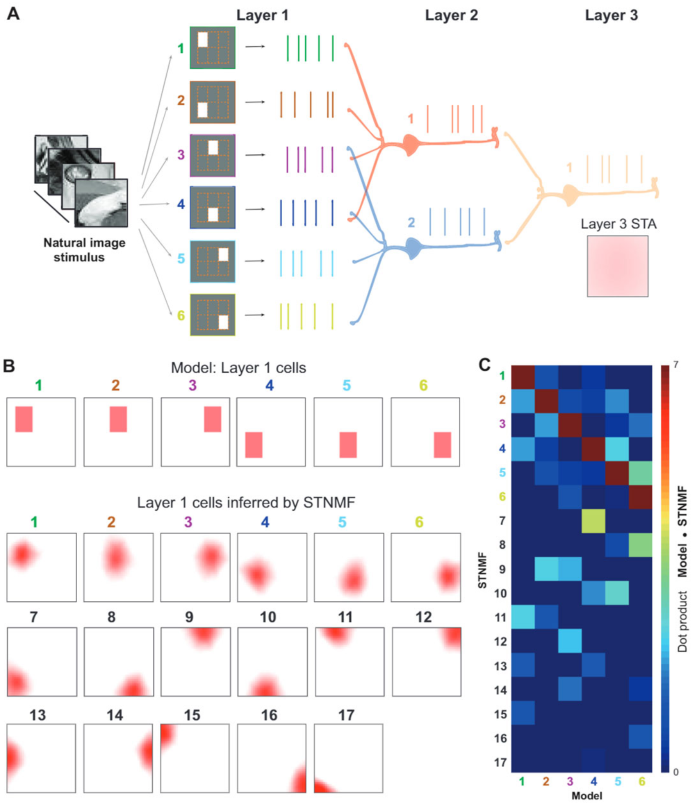

Fig. 4 STNMF works well for natural image stimulus.

QII-4. minor points: in several places, LGN was mistyped by LNG

AII-4: We have revised it.

****

Replies to the Comments from Reviewer #3

****

The authors applied non-negative matrix factorization (NMF) to the analysis of neuronal networks. They tested the method on model circuits that are similar to the early visual systems by inferring information regarding the circuit using the spike trains of postsynaptic neurons.

The core idea is that the stimuli (movies) are first convolved with the temporal STAs at each pixel. These convolved images at the time of spikes were subjected to NMF to give two matrices representing the effective inputs from individual modules and the spatial patterns of those modules. The authors showed that the method successfully estimated the stimulus-response properties of the presynaptic neurons and the synaptic weights for network models including three-layer models.

The method is interesting and would be useful for some kinds of network analyses. For the paper to be fully useful for the neuroscience community, I recommend improving the following points.

QIII-1. Determination of the number of modules. For NMF to be successful, determining the number of modules (K) is critical. If my understanding is correct, authors appear to assume that K is given. However, this is unlikely in the analysis of real neuronal responses. Please discuss this point or include a method to estimate K.

AIII-1: Thanks for the comment. We have clarify this point. See our response **A1-1** above.

QIII-2. Overlap of spatial filters. In all the circuit models shown in the paper, the spatial filters of RGCs with the same sign (ON or OFF) do not overlap. This is unlikely in real neuronal circuits. In addition, because the method uses pixel-wise temporal convolution to generate the matrix S, the fact that the spatial filters do not overlap may have contributed to the results. Please discuss whether the method works when RGCs with the same sign spatially overlap.

AIII-2: Thanks for the comment. We have clarified this point. See our response **A1-2** above.

QIII-3. The number and density of presynaptic neurons. The models in the paper contain relatively small number (≦10) of presynaptic neurons. Although this is reasonable for method evaluation and for certain neuronal circuits, many neurons receive non-trivial inputs from a much larger number of presynaptic neurons. Please discuss whether the method works for such networks.

AIII-3: Thanks for the comment. We agree with the reviewer that many neurons receive non-trivial inputs from a much larger number of presynaptic neurons. There is a rich picture about cell types and spare firing of synapses and neurons, see our response **A1-3** for detail and we added a new subsection in Discussion about this point (red text at line 394 'A. The role of presynaptic neurons in postsynaptic neural spikes').

STNMF essentially can scale up to a large number of synaptic inputs, as long as nonlinear computation is preserved by each synapse in the network. For the cost of computational resources, we designed a simulation with 16 neurons in the first layer. The analysis of STNMF reveals that it works well with more presynaptic neurons, see below and Supplemental Fig. S2 in the revised manuscript.

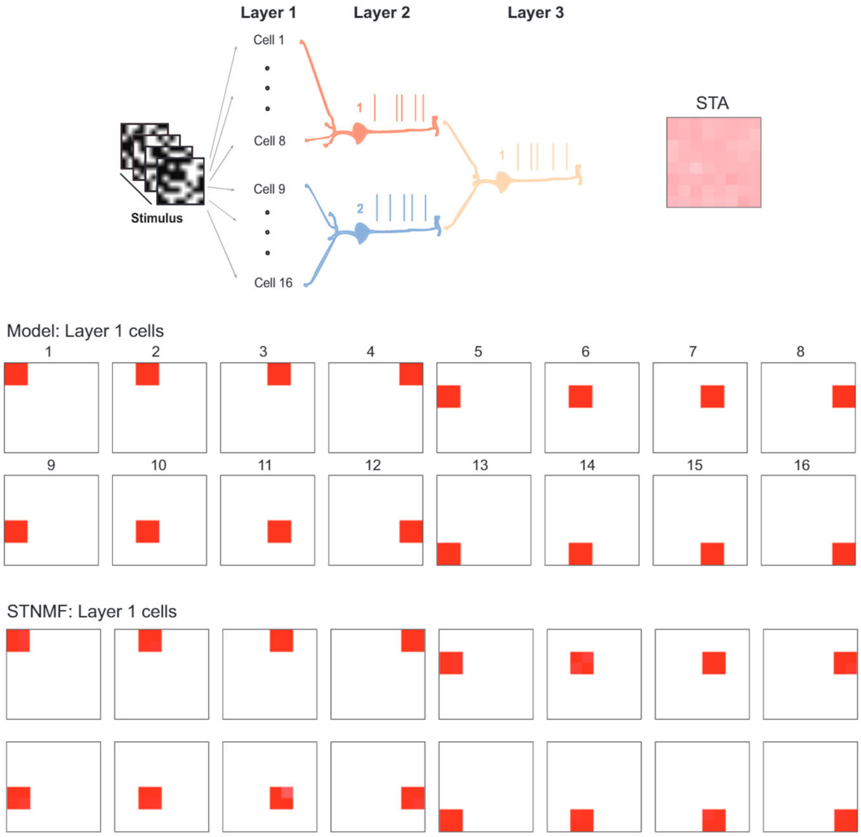

Fig. S2. STNMF for more cells.

Below are minor points:

QIII-4. Line 104: “Wi” should be in lowercase.

AIII-4: We have revised it.

QIII-5. The manuscript appears to contain non-standard mathematical notations. For example, Line 126: In the notation for the weighted average, a dot seems to represent the dot product, but this is not clearly stated.

AIII-5: Thanks for pointing this out. We have revised it and clearly check all other math notations.

QIII-6. Line 138: The suffix i appears unnecessary. Please carefully check mathematical notations.

AIII-6: We have revised it and clearly check all other math notations.

Q: Reviewer #2: **No:** it will be better if authors provide data samples and computational codes for audients to reproduce their figures.

A: Yes, we will make our code available to the public as part of publication at the dedicated Data Availability statement:

The code used to generate the results in this paper will be available on https://github.com/jiankliu/STNMF-SNN.
